# Novel insights into the insect trancriptome response to a natural DNA virus

**DOI:** 10.1186/s12864-015-1499-z

**Published:** 2015-04-17

**Authors:** Seanna J McTaggart, Tidbury Hannah, Stephen Bridgett, Jennie S Garbutt, Gaganjot Kaur, Mike Boots

**Affiliations:** Institute of Evolutionary Biology, School of Biological Sciences, Ashworth Laboratories University of Edinburgh, Edinburgh, EH9 3JT UK; Centre for Immunity, Infection and Evolution, School of Biological Sciences, Ashworth Laboratories University of Edinburgh, Edinburgh, EH9 3JT UK; Centre for Environment, Fisheries and Aquaculture Science, The Nothe, Barrack Road, Weymouth, Dorset, DT4 8UB UK; Edinburgh Genomics, Institute of Evolutionary Biology, School of Biological Sciences, Ashworth Laboratories University of Edinburgh, Edinburgh, EH9 3JT UK; The Centre for Applied Genomics, The Hospital for Sick Children, Toronto, ON M5G 1X8 Canada; Daphne du Maurier Centre for Ecology and Conservation, College of Life and Environmental Sciences, University of Exeter, Cornwall Campus, Cornwall, TR10 9EZ UK

**Keywords:** Differential gene expression, DNA virus, de novo transcriptome assembly, host-pathogen interaction, RNA-Seq

## Abstract

**Background:**

Little is known about invertebrate responses to DNA viruses. Here, we infect a commercially important pest moth species *Plodia interpunctella* with its naturally infecting DNA virus. We sequenced, assembled and annotated the complete transcriptome of the moth, and a partial transcriptome of the virus. We then tested for differential gene expression between moths that were exposed to the virus and controls.

**Results:**

We found 51 genes that were differentially expressed in moths exposed to a DNA baculovirus compared to controls. Gene set enrichment analysis revealed that cuticle proteins were significantly overrepresented in this group of genes. Interestingly, 6 of the 7 differentially expressed cuticle proteins were downregulated, suggesting that baculoviruses are able to manipulate its host’s response. In fact, an additional 29 of the 51 genes were also downregulated in exposed compared with control animals, including a gram-negative binding protein. In contrast, genes involved in transposable element movement were upregulated after infection.

**Conclusions:**

We present the first experiment to measure genome-wide gene expression in an insect after infection with a natural DNA virus. Our results indicate that cuticle proteins might be key genes underpinning the response to DNA viruses. Furthermore, the large proportion of genes that were downregulated after viral exposure suggests that this virus is actively manipulating the insect immune response. Finally, it appears that transposable element activity might increase during viral invasion. Combined, these results provide much needed host candidate genes that respond to DNA viral invaders.

## Background

Hosts are constantly challenged by the ubiquitous presence of pathogens and understanding the genetic architecture of these interactions is critical to developing tools to prevent infection. Within insects, characterization of innate immune system genes and pathways has primarily focused on responses to bacterial and fungal pathogens [1]. In contrast, the genes that respond to viruses are less well understood, with the vast majority of research limited to work with RNA viruses [2]. Our understanding of insect immune responses to DNA viruses is poor and is a critical void that needs to be filled. Two studies that have quantified gene expression in *Drosophila* after exposure to a DNA virus (Invertebrate iridescent virus 6) demonstrated that the RNA interference (RNAi) pathway can be effective at removing DNA as well as RNA viruses, although how this occurs is unknown [3,4]. In addition, the evolutionarily conserved immune pathway JAK-STAT may play a role in both DNA and RNA viral defence, but only against a subset of viruses [4].

However, these two studies, like many of the functional studies elucidating the roles of invertebrate immune system genes, do not challenge the host with a natural pathogen. Since the immune response to a novel pathogen is unlikely be equivalent to the product of an antagonistic co-evolutionary relationship, it is imperative to also probe the immune response in naturally-occurring host-parasite systems. Additionally, these studies were carried out in a single species of *Drosophila* and therefore the generality of their findings are unclear. Finally, comparative genomics of fully sequenced insect genomes reveals that not all immune system genes are present in all taxonomic groups. For example, the pea aphid, *Acyrthosiphon pisum*, does not have key genes involved in recognition and signalling in the IMD immune pathway [5]. Thus, in order to build a comprehensive understanding of innate immune systems, it is important to survey a wide variety of taxonomic groups exposed to different pathogen types. Given our lack of understanding of the immune responses to DNA viruses, it is particularly important to examine natural DNA viral infections of non-model hosts.

To this end, we exposed a moth species, *Plodia interpunctella*, to its naturally infecting DNA virus *Plodia interpunctella* Granulosis Virus (PiGV). *P. interpunctella*, the Indian meal moth, is a major pest of stored dry food products around the world, causing significant economic losses [6]. We sequenced the complete transcriptomes of exposed and control moths using RNA-Seq (Illumina), and assembled them using and comparing two different commonly used assemblers (SOAP*denovo-Trans* [7] and Trinity [8]). The chosen transcriptome assembly of the moth and a partial transcriptome assembly of the virus are available as a public resource at http://afterparty.bio.ed.ac.uk/. Finally, we characterized the virally-induced transcriptome of the moth, therefore adding much needed information on the genetic architecture of insect- DNA virus interactions.

## Results

### De novo transcriptome assembly assessments

After filtering for high quality sequences, a total of 488,010,769 sequences (per sample average = 27,111,709 sd 5,923,264) were used to construct four assemblies using alternate methods. All samples were used to construct each assembly. Overall, the assemblies constructed with Trinity had a greater maximum contig length than SOAP*denovo-Trans* (62,936 bases versus 46,673 bases), while the SOAP*denovo-Trans* assembly had a larger median contig length than the Trinity assembly (428 bases versus 393 bases) (Table 1). Both of the Trinity assemblies had many more contigs than either of the SOAP*denovo-Trans* assemblies (135,990 contigs versus 82,753 contigs), which were longer (32,900 contigs > =1 kb versus 20,725 contigs > =1 kb). Likewise, many more bases were included in the Trinity assemblies compared to the SOAP*denovo-Trans* assemblies (140,975,202 compared to 85,762,961 bases, respectively).

**Table 1:** **Transcriptome assembly statistics**

	**SOAP** ***transdenovo***		**Trinity**	
	**kmer size =25**	**kmer size=31**	**Edge threshold =0.05**	**Edge threshold =0.16**
Max contig length	46,673	46,631	62,936	62,936
Mean contig length	1,053	1,036	1,037	905
Standard deviation of contig length	1,595	1,559	1,736	1,542
Median contig length	424	428	393	375
N50 contig length	2,539	2,452	2,617	2,125
Number of contigs	80,425	82,753	135,990	127,665
Number of contigs>=1kb	20,249	20,725	32,900	26,818
Number of contigs in N50	8,933	9,330	14,198	13,689
Number of bases in all contigs	84,708,481	85,762,961	140,975,202	115,544,987
Number of bases in contigs>=1kb	60,770,939	60,961,055	101,519,875	77,125,576
GC Content of contigs	38.82%	38.95%	39.35%	38.96%

There were very minor differences in the number of reads that mapped to each of the assemblies (Table 2). Over 90% of the reads mapped back to each of the four assemblies, with the most (98%) mapping to the Trinity assembly (Edge threshold = 0.16). They all had equivalent numbers of uniquely mapping reads (which was the only category of mapped reads that was considered in the analysis of differential expression) ranging from 68-74%.

**Table 2:** **Mapping statistics**

	**SOAP** ***transdenovo***				**Trinity**			
	**kmer size =25**	**%**	**kmer size=31**	**%**	**Edge threshold =0.05**	**%**	**Edge threshold =0.16**	**%**
Total # of fragments	376,604,607		376,604,607		376,604,607		376,604,607	
Total # of mapping fragments	345,067,834	91.63	356,499,243	94.66	351,206,829	93.26	368,474,636	97.84
# of chr in reference	80,425		82,753		135,990		127,665	
**Uniquely mapping pairs**								
Concordant	236,768,017	68.61	252,262,424	70.76	240,006,041	68.34	260,088,477	70.59
Halfmapping	10,181,639	2.95	10,833,441	3.04	4,222,926	1.20	3,128,004	0.85
unpaired	1,674,474	0.49	2,975,582	0.83	758,404	0.22	925,244	0.25
**Multiply mapping pairs**								
Concordant	93,559,753	27.11	87,279,976	24.48	103,451,332	29.46	100,980,778	27.41
halfmapping	2,102,511	0.61	2,270,699	0.64	1,287,909	0.37	1,710,051	0.46
unpaired	581,342	0.17	611,289	0.17	1,240,292	0.35	1,256,968	0.34
**Translocated read pairs**								
concordant	0		0		0		0	
Halfmapping	0		0		0		0	
unpaired	0		0		0		0	
**Others**								
paired multimapping	35,508	0.01	33,876	0.01	48,569	0.01	53,351	0.01
paired unique inverted	116,861	0.03	84,905	0.02	65,638	0.02	71,371	0.02
paired unique long	25,618	0.01	50,100	0.01	7,667	0.00	8,165	0.00
paired unique scr	22,111	0.01	96,951	0.03	118,051	0.03	252,227	0.07
No mapping	31,536,773	9.14	20,105,364	5.64	25,397,778	7.23	8,129,971	2.21

Prior to searching each of the transcriptomes for ultra-conserved orthologs (UCO), we used USearch [9] to collapse contigs that differed by less than 3% sequence divergence from the chosen Trinity assembly. This resulted in ~ 9% reduction in the number of contigs. More of the UCO were recovered with the Trinity assemblies compared to the SOAP*denovo-Trans* assemblies (Table 3), and more genes were only found once within the Trinity versus the SOAP*denovo-Trans* assemblies. The Trinity assembly with the parameter edge threshold = 0.16 recovered slightly more of the UCOs only once, compared to the assembly with an edge threshold of 0.05, and thus the former was chosen for the differential expression analysis. After analysis with the EviGene pipeline, the contig set was reduced to N = 18,475. However, to ensure that all putative transcripts were made publically available, the full (i.e. uncollapsed) transcriptome was used as the input to afterParty [10] and the reduced transcriptome is available as a subset of these data.

**Table 3:** **The number of recovered conserved orthologs found in each of the four** ***de novo Plodia interpunctella*** **transcriptomes**

	**SOAP kmer25**		**SOAP kmer31**		**Trinity 0.05**		**Trinity 0.16**	
**Number of copies**	**Original**	**USearch**	**Original**	**USearch**	**Original**	**USearch**	**Original**	**USearch**
0	28	28	26	26	21	21	21	21
1	153	180	174	205	175	209	191	216
2	104	103	91	89	83	90	85	90
3	37	25	35	18	27	16	23	13
4	16	13	17	12	19	11	15	8
5 or more	19	8	14	7	32	10	22	9

The search was conducted on N=357 ultra-conserved orthologs. A count is given for the orginal transcriptome build and for the build after clustering with USearch.

### Functional annotation

**Figure 1:**
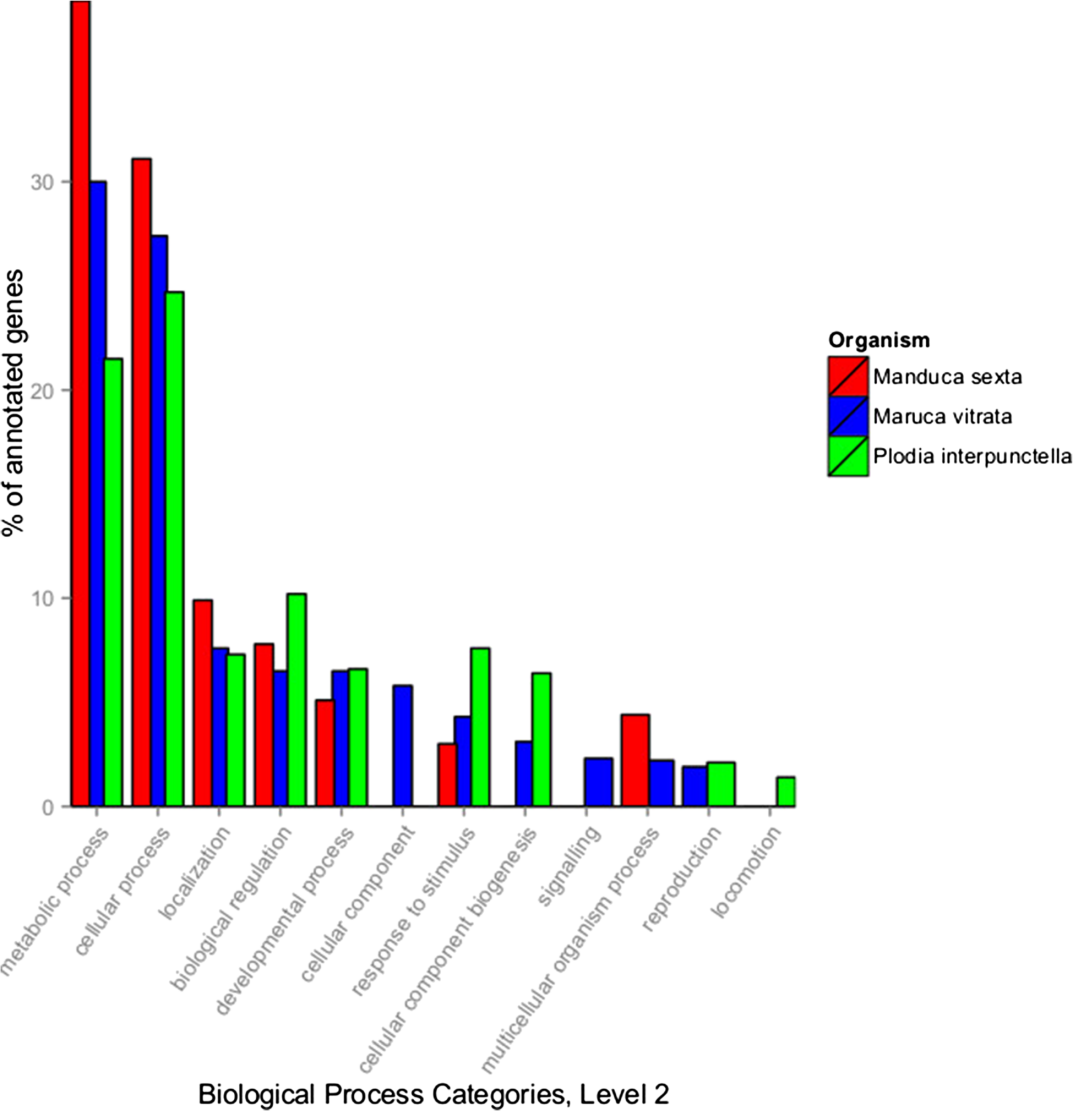
Gene ontologies assigned to three insect species by the Gene Ontology database. All assignments are for biological process at GO level 2. Data for *Maruca vitrata* was from [10], data for *Manduca sexta* was from [11] and data for *Plodia interpunctella* was from this study. Note that one sequence can be assigned to more than one GO term.

A total of 5396 (29%) contigs were not homologous with any sequences present in the NBCI non-redundant database. Of the remaining 13,079 contigs that had a BLAST result, 9917 were not functionally classified. The remaining 3162 contigs were successfully annotated with GO terms. From the functionally annotated contigs, putative genes from the RNAi, IMD, Toll and JNK pathways were identified, although not all genes within these pathways were present (Table 4). The biological process categories (level 2) that contained the highest percentage of annotated genes were (1) cellular process (24%) and (2) metabolic process (22%). These two categories also represent the two largest sets of genes in the transcriptomes derived from the whole body of the pod borer, *Maruca vitrata* [11]*,* as well as that of the midgut of the insect *Manduca sexta* [12] (Figure 1). However, a chi-square test shows that the distributions of GO terms between the three species’ transcriptomes (p < 0.001) are different. Additionally, fewer GO terms were identified in the *M. sexta* data set than in *P. interpunctella*, however, this is likely because the transcriptome of *M. sexta* was derived from a specialized tissue (the midgut).

**Table 4:** **Presence or absence of immune related genes in lepidopteral** ***Plodia interpunctella***

**Gene**	**Pathway**	**Elicited by**	**Present**
Dicer	RNAi	viruses	yes
R2D2	RNAi	viruses	yes
Argonaut	RNAi	viruses	yes
Aubergine	RNAi	viruses	yes
vig	RNAi	viruses	No
Armi	RNAi	viruses	yes
Drosha	RNAi	viruses	yes
PGRP	IMD	Bacteria	yes
IMD	IMD	Bacteria	No
dFADD	IMD	Bacteria	No
Dredd	IMD	Bacteria	No
dTAK	IMD	Bacteria	No
IKKy	IMD	Bacteria	yes
IKKb	IMD	Bacteria	yes
Relish	IMD	Bacteria	yes
Sick	IMD	Bacteria	No
Dnr1	IMD	Bacteria	No
JNK	JNK	Bacteria	yes
Hep	JNK	Bacteria	Yes
Bsk	JNK	Bacteria	No
Nimrod	receptors		No
DSCAM	receptors		yes
Hemes	receptors		No
GNBP	PAMP	Bacteria	yes
Persephone	Toll	Fungi, Bacteria	No
serine proesases	Toll	Bacteria	Yes
Spatzle	Toll	Bacteria	Yes
Toll	Toll	Bacteria	yes
Pelle	Toll	Bacteria	yes
MyD88	Toll	Bacteria	yes
Tube	Toll	Bacteria	No
Cactus	Toll	Bacteria	yes
Dorsal	Toll	Bacteria	yes
Dif	Toll	Bacteria	No
prophenoloxidase	Toll	Bacteria	Yes
antimicrobial peptides	Toll,IMD/JNK	Bacteria	yes
Domeless	JAK/STAT	viruses	No
JAK	JAK/STAT	viruses	Yes
STAT	JAK/STAT	viruses	Yes
TEP	JAK/STAT	viruses	Yes

All putative genes can be found at http://afterparty.bio.ed.ac.uk/study/show/2194070.

**Figure 2:**
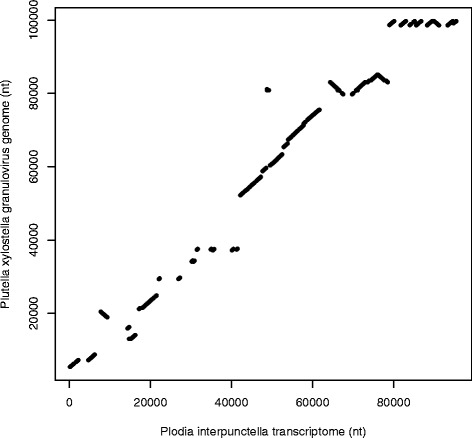
Nucleotide sequence similarity dot plot of the *de novo* transcriptome of *Plodia interpunctella* granulovirus (this study) versus the assembled *Plutella xylostella* granulovirus genome (NCBI reference sequence NC_002593.1). Nucleotide identity between the two sequences is represented by a dot. This analysis suggests that the current *P. interpunctella* granulovirus transcriptome assembly is approximately 44% complete.

A total of 27 contigs spanning 95,556 nucleotides were likely of PiGV origin. A dot plot against *Plutella xylostella granulovirus* (NCBI reference sequence NC_002593.1), suggests that this assembly covers about 44% of the viral genome (Figure 2). This contig set is also available at the afterParty website (http://afterparty.bio.ed.ac.uk). Surprisingly, some of the genes recovered include genes expressed in late and very late infection stages, such as those involved in viral replication (DNA polymerase, DNA ligase, and a helicase) and transmission (envelope fusion protein and envelope fusion protein) [13] as well as a chitinase.

### Infection outcome and differential expression

**Figure 3:**
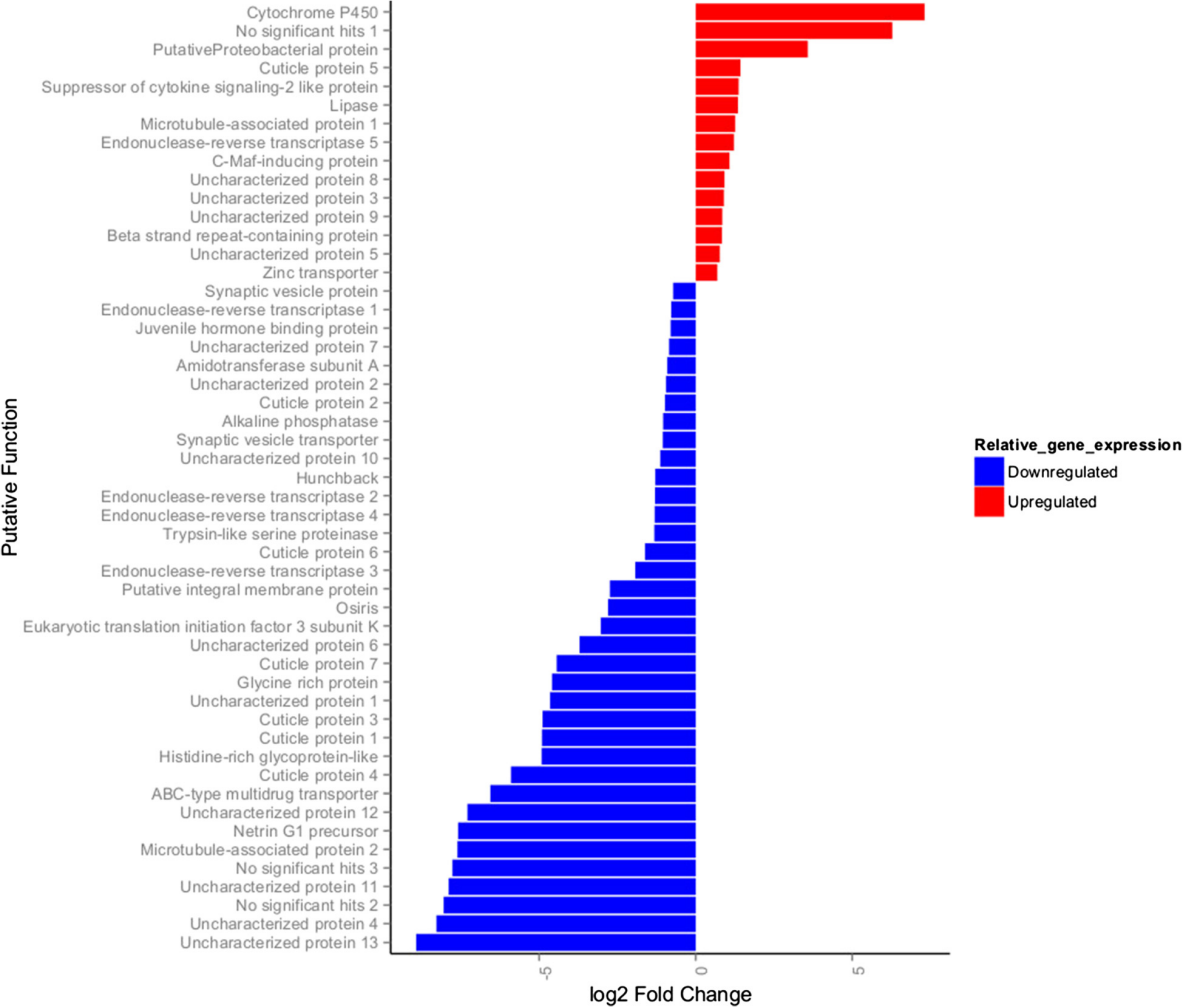
Log2 fold changes in 51 differentially expressed genes in *Plodia interpunctella* 24 hours after exposure to a virus compared to controls. Gene expression level was estimated by counting the number of uniquely mapped sequencing reads to each gene. Differential expression was calculated with DESeq ([25]), with a FDR threshold of <0.10. Blue bars indicate genes are downregulated and red indicates that genes are upregulated in exposed versus control moth larvae.

On average, 77.6% (sd = 7%) of the larvae that were exposed to the viral solution became infected. No larvae that were exposed to the control treatment were infected. Fifty-one genes were significantly differentially expressed 24 hours after exposure to PiGV, the majority (N = 36) of which were downregulated (Figure 3). These 51 genes have many different functions, including one canonical immune system gene (a homolog of the gram-negative binding protein (GNBP) Osiris), 7 cuticle proteins, a juvenile-hormone binding protein, 5 genes potentially involved in transposition and 13 genes of unknown function. Gene set enrichment analysis determined that only the cuticle proteins were enriched in this data set (FDR = 1.8E-2). Interestingly, not all genes of the same putative function were regulated in the same way: six of the cuticle proteins were virally downregulated while one of them was virally up-regulated.

One of the 51 differentially expressed genes was not of the host origin. Indeed, its closest homolog was a putative proteobacteria. This gene was very lowly expressed in virally exposed larvae and absent in controls. Additionally, 3 of the differentially expressed genes have no known similarity to any other proteins, thus it was not possible to determine whether they were of host, viral or some other origin.

### q-RT-PCR

**Figure 4:**
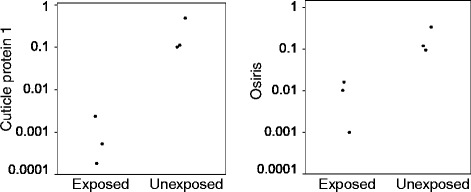
Confirmation of expression using q-RT-PCR. Expression of comp623_c0_seq1 (cuticle protein 1) and comp2004_c0_seq1 (Osiris (a putative GNBP)) relative to actin (comp24_c0_seq1) in the two infection treatments.

We analysed the expression of comp623_c0_seq1 (cuticle protein) and comp2004_c0_seq1 (GNBP) using comparative C_T_ (ΔΔC_T_) qPCR. The expression of both genes was significantly lower in moths that were exposed to the virus (Figure 4, note the log scale; comp623_c0_seq1 t_1,4_ = 6.34, p = 0.003; comp2004_c0_seq1 t_1,4_ = 3.55, p = 0.02).

## Discussion

### Assembly and functional annotation

Two *de novo* transcriptomes were assembled from each of SOAP*denovotrans* and Trinity. Overall, the Trinity assemblies contained more and longer contigs than the SOAP assemblies, and seemed to have slightly better coverage of highly conserved, single copy orthologous genes. In addition to recovering 95% of the ultra-conserved orthologs (60% of which were present as a single copy), gene candidates were identified from all canonical immune gene pathways (e.g. RNAi, Jak/STAT, Toll, IMD), supporting the relative completeness of the assembly. As expected for biological samples that were not controlled for life-history stage, experimental conditions or tissue type, when broken down into functional categories through the use of GO terms, the *P. interpunctella de novo* assembly differs from other lepidopteran transcriptomes (Figure 1).

### Differential expression

Forty-seven *P. interpunctella* genes were differentially expressed 24 hours after exposure to PiGV compared to exposure to a control solution. The observed changes in expression could have several mechanistic origins, including defence, tolerance and repair. The differentially expressed genes were enriched for cuticle proteins (N = 7), 6 of which were virally downregulated and 1 was upregulated. To our knowledge, cuticle proteins have not been implicated previously from studies examining mRNA levels in cell cultures after infection with a baculovirus [14]. Cuticle proteins could play a role in defence in at least the following three ways. First, cuticle proteins form a large proportion of the peritrophic membrane (PM). The PM lines the gut and provides the first line of defence against ingested pathogens, such as PiGV and has been strongly implicated in antiviral defence [15,16]. For example, the PM of more susceptible velvetbean caterpillars, *Anticarsia gemmatalis* had a lower chitin content and provided a less efficient barrier against its baculovirus (AgMNPV) than more resistant larvae [15]. Furthermore, changes in the peritrophic membrane are correlated with changes in the risk of pathogen infection. For example, the thickness of the PM is also well known in *Anopheles* mosquitoes to increase after the ingestion of a blood meal, which is the primary source of infective pathogens [16].

The change in expression of cuticle proteins in *P. interpunctella* could reflect similar processes. Generally, cuticle proteins in the peritrophic membrane have a distinctive molecular signature, which is not present in any of the significantly differentially expressed cuticle proteins identified. However, since studies characterizing PMs have only been conducted in a limited number of species, which are not closely related to the lepidopteran *P. interpunctella*, it is possible that the molecular signature is too divergent to recognize. Furthermore, not all proteins of the PM have been characterized. For example, [17] recently identified a new PM protein in the meadow moth that is able to bind chitin, but does not contain the conserved binding domain. Secondly, expression of cuticle proteins has been monitored in insects and been shown to change when moulting takes place [18]. In *Plodia*, moulting includes shedding the gut lining, to which the PiGV particle may attach prior to penetration into the haemolymph. Thus, the differentially expressed cuticle proteins might correspond to *Plodia* moulting. Finally, the cuticle proteins may directly inhibit viral replication. For example, [19] demonstrated that a mosquito cuticle protein (AAEL011045) binds to a viral envelope and thus inhibits infection. Furthermore, they discovered that this protein was *down*regulated in virally exposed mosquitoes, raising the possibility that the virus was actively suppressing the expression of this molecule.

**Figure 5:**
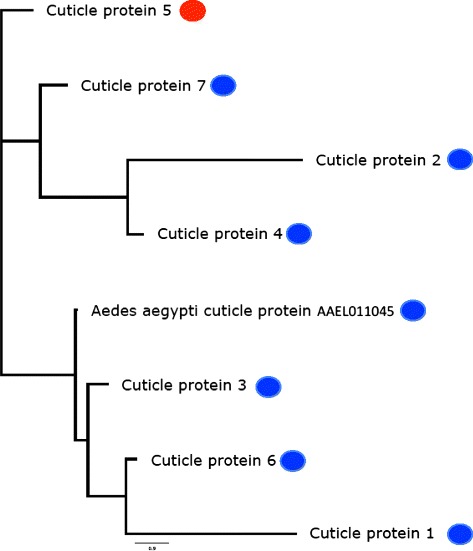
Neighbour-joining tree of 7 differentially expressed cuticle proteins from *Plodia interpunctella* and a cuticle protein from *Aedes aegypti*. All genes marked ‘cuticle protein’ are of *P. interpunctella* origin. Genes marked with a blue dot are downregulated after exposure to a pathogen, will the gene with a red dot (cuticle protein 5) was upregulated after exposure to a pathogen.

In order to discover if the degree of sequence identity amongst the 7 differentially expressed cuticle-proteins in this study could be used to explain their opposing pattern of regulation (i.e. up versus down), we aligned them with mosquito cuticle protein AAEL011045 in BioEdit v.7.2.4 [20] and built a neighbour-joining tree using CLUSTAL v1.2.0 [21] (Figure 5). This phylogenetic analysis shows that the seven moth genes cluster into three major clades; two clades of three genes each and a singleton. The singleton is the only cuticle protein that is upregulated after viral exposure. The moth genes in the clade containing the mosquito cuticle protein AAEL011045 are all candidate immune system genes that may be able to bind to the envelope of PiGV and thus inhibit its infectivity.

Two of the most up-regulated genes after viral exposure are a reverse transcriptase and a transposase. This suggests that within 24 hours of exposure to a virus, transposable elements (TE) activity, or ‘jumping’, is switched on (there were no reads from the control samples that mapped to either of these two genes). Supporting this hypothesis, a study that detected differential expression in hemocytes of the moth larvae *Heliothis virescens* after infection with *Helicoverpa zea* single nucleopolyhedrovirus found many retrotransposons to be upregulated [22]. A recent study in *Drosophila* showed that fragments of a virus were reverse-transcribed, producing truncated versions of the virus, which were processed by the RNAi machinery resulting in a reduction in the amount of active virus in the cells [23]. Due to the increase in transposase activity found in this system, it will be interesting to test if there is a connection between TE activity and the control of a viral infection in this system as is the case in *Drosophila*.

Of all of the differentially expressed genes, 36 (70%) were downregulated. The prevalence of down-regulation suggests that PiGV may directly or indirectly exert a considerable inhibitory effect on the host immune response. Indeed, late in infection, some studies in cell culture have documented a global downregulation of host genes (e.g. [24]). Similar effects of viral suppression on host immunity have been documented in other *in vivo* insect/viral systems (*Aedes aegypti* infected with Dengue-virus [19,25], West Nile Virus and Yellow Fever Virus [19]). Indeed, in our differentially expressed data set, the only canonical immune system gene, a gram-negative binding protein (GNBP), is downregulated in virally-exposed larvae compared to control larvae. GNBPs are intricately involved activating the Toll pathway, and are upregulated after exposure to bacterial pathogens. The fact that the GNBP identified in this experiment is downregulated suggests that the virus may be suppressing the TOLL pathway. This potential for a trade-off between the immune response to bacteria versus viruses has been documented in mosquitoes, where the growth of *E. coli* was enhanced after viral infection with dengue virus (an RNA virus) in *A. aegypti* hosts [25]. They went on to show that viral interference with the host immune system resulted in a decreased production of antimicrobial peptides. Our transcriptome assembly contains several putative antimicrobial peptides, but none of them were differentially expressed after infection with the virus. However, our sampling time point was very early in the infection process, and thus sampling at more time points will be necessary to test if the viral infection has an effect on antimicrobial peptide activity.

The change in GNBP transcription may support the hypothesis that the innate immune response to a viral infection is not be restricted to the RNAi pathway, but instead additionally involves the Toll pathway. However, it is difficult to distinguish if the activation of different immune pathways is a direct or indirect result of the virus. For example, if the virus crossing the gut membrane results in a wound, naturally occurring gut microbes will also pass into the haemolymph and trigger an immune reaction. Our finding that one of the differentially expressed genes in the moth larvae was of bacterial origin is consistent with this hypothesis. Because we only assessed transcription very early in infection, it is not possible to determine if other members of the Toll pathway would also be differentially expressed.

Some of the genes that were determined to be differentially expressed are not functionally annotated (N = 16, 31%). Many of these genes have high sequence similarity to other organisms, suggesting that functionally conserved, important putative immune, tolerance or repair genes have yet to be characterized in insects. Additionally, some of the genes had no known homology to other organisms, suggesting that there are also highly specific genes that are functionally uncharacterized. This study provides a solid foundation for choosing candidates for further functional characterization.

## Conclusions

We have identified many candidate genes involved in the molecular response of a moth species to a naturally infecting DNA virus, a response for which we previously had a very limited understanding. Our results support a growing body of evidence that non-canonical immune system genes such as cuticle-proteins may play a significant role in the insect response to a range of pathogens. Additionally, transposition rates of mobile elements may be significantly altered during a viral attack.

Furthermore, this study represents the first comprehensive mRNA sequencing effort in this economically important pest species. The sequences from this study provide an important resource for studies of molecular genetics and functional genomics of *Plodia interpunctella*. For example, these data can be used to develop microarrays for gene expression analysis or to serve as a reference transcriptome for future RNA-seq experiments with *P. interpunctella*. Finally, we have also assembled a portion of the viral genome, which will allow molecular markers to be developed, which can, for example, aid in assessing the prevalence of this virus in natural populations.

## Methods

### Larval culture, RNA extraction, library preparation and sequencing

The host species used in our experiment was *Plodia interpunctella*, the Indian meal moth. All individuals were taken from a large outbred stock that has been maintained at the University of Sheffield for approximately 8 years and reared on a cereal-based diet under standard laboratory conditions (27°C; 16 L:8D cycle). The pathogen used was *Plodia interpunctella* Granulosis Virus (PiGV), a natural DNA virus of *P. interpunctella*. The virus is naturally transmitted through the ingestion of virus occlusion bodies either from the environment or through necropsy of infected individuals. Baculoviruses (BVs) are generally host-specific, obligate killing DNA viruses [26] with two phenotypes; the occluded virus, which consists of virions encased within a protein coat, and the budded virus. The initial site of virus exposure in the insect is the midgut where the alkaline conditions destroy the occlusion body resulting in the release of infectious virions. These virions may cross the peritrophic membrane into the epithelial cells and systemic infection is established when progeny BVs pass across the basal lamina into the haemocoel and infect secondary tissue such as the tracheal system and the fat body [27]. Our aim was to capture the change in mRNA levels during early infection, as immune responses can be swift and transient [28]. To this end, we chose to sample at 24 hours post infection, at which point enveloped virus nucleocapsids were found trapped in the connective tissue surrounding the midgut [29].

Transcriptome assemblies were constructed from 18 *P. interpunctella* samples sequenced with RNA-Seq (Illumina) 100 base, pair-end reads on three HiSeq lanes (Illumina). The 18 samples came from two independent experiments, one of which (N = 12) is described in a separate paper (McTaggart et al., *in prep*). Each sample consisted of 20 pooled individuals. Here, our aim was to characterize the transcriptomes of larvae, 24 hours after they had been exposed to viral dose of LD_50_. We set up six replicates in each of two treatments (viral exposed and control). In each replicate, 30 F1 generation adult moths were kept together with excess food. Prior to their viral exposure, 50 fourth instar larvae were removed from the pot, starved for 2 hours and then inoculated with the virus using a standard droplet feeding method [27]. The virus solution was prepared by centrifugation of infected *P. interpunctella* larvae, and diluted in blue food colouring and sucrose. The required dose was calculated from a dose response assay performed prior to the experiment.

We inoculated larvae with either a solution of purified PiGV to LD_50_ or with a control solution consisting of only sucrose and blue food colouring. Both treatments were orally administered with a standard oral droplet feeding protocol, and considered successful if the blue solution was visible in at least half of the gut length. Inoculated larvae were moved to individual wells of a 25-cell Petri dish containing ample food. Twenty-four hours later twenty larvae from each replicate were pooled and crushed in 1 ml of Trizol (Life Technologies) and stored at −20°C until RNA extraction. The remaining larvae (N ~ 30) were monitored for infection for 30 days. Due to financial constraints, only three of the six replicates from each treatment (i.e. six samples in total) were sequenced using RNA-Seq.

Immediately before RNA extraction, an additional 500ul of TRIzol (Ambion, Life Technologies) was added to each sample, and incubated for 5 minutes at room temperature. Two hundred ul of chloroform was added to each sample, shaken vigorously for 15–20 seconds and then centrifuged at 11600 rcf for 15 minutes at 4°C. The upper, aqueous phase was isolated and nucleic acids precipitated by adding 0.5 volumes of Absolute Ethanol, and inverting the tubes several times. This solution was used as the starting material for the RNAeasy (Qiagen) protocol, which was followed according to the manufacturer’s instructions. The integrity of the resultant total RNA was confirmed on Bioanalyzer (Agilent RNA-nano reagents). RNA and DNA concentrations were determined with a Qubit fluorometer (Invitrogen QuantRNA), while the 260:280 ratio was assessed on a Nanodrop (ThermoScientific). For each sample, we subjected 5 ug of total RNA to one round of poly-A selection on oligo(dT) Serabeads. The resultant messenger RNA was fragmented to an average size of 100 bp using divalent cations at 95°C for 5 min prepared following the manufacturer’s recommended protocol (Illumina mRNAseq kits Cat no. RS-100-0801). First strand cDNA synthesis was carried out using Superscript III reverse transcriptase (Invitrogen) and 3 ug random hexamer primers (Illumina) per sample as per the manufactures’ instructions. Second strand cDNA synthesis and RNAseq samples were prepared according to the manufacturer’s recommended protocol (Illumina). The fragment size and concentration of resultant libraries were assessed on a Qubit fluorometer (Invitrogen QuantRNA) and on a Bioanalyser High Sensitivity Chip (Invitrogen QuantRNA).

### Transcriptome assemblies

Adapter sequences were trimmed from the raw reads using the program Scythe (https://github.com/vsbuffalo/scythe). The program Sickle (https://github.com/najoshi/sickle) was used to remove low quality bases, reads with N’s, and sequences that were less than 50 bases long. SOAP*denovo-Trans* [7] was used to build two assemblies with different k-mer sizes (kmer = 25, kmer = 30). Otherwise, default parameter settings were used. The assembler Trinity [8] was also run on the data twice, once with an edge threshold parameter of 0.05 and the other of 0.16. Default settings were used for all of the other parameters. Reads were mapped back to each assembly using GSNAP (http://research-pub.gene.com/gmap/). Reads thought to be due to PCR duplication were flagged with Picard (http://broadinstitute.github.io/picard/) and were not considered in any analyses. All contigs that had three or fewer reads mapping to it across all treatments were considered to be misassembled and removed.

In order to determine if any of the assembled contigs were of viral origin, the complete genome of *Plutella xylostella granulovirus* (NC_002593.1)(PxGV) was queried against the *P. interpunctella* transcriptome. Any contig that had a BLAST hit with an e-value of less than 1e-30 was considered to be viral. A dot-plot of these transcripts was made against the PxGV genome to estimate how much of the PiGV transcriptome had been recovered.

### Assembly assessment

For the purposes of comparison, we assessed three basic parameters of each of the four assemblies: contig length, the number of contigs and the number of bases in the contigs. We also quantified the proportion of the sequence reads that mapped to each assembly using GSNAP. To test the completeness of the assemblies, we tested for the presence of highly conserved genes from other species in two ways. First, we built a BLAST database of each transcriptome that was queried with a previously published set of ultraconserved single-copy orthologs (UCOs) using tBLASTn. We counted how many times each gene was observed (with the expectation that each gene should be present only once) in each transcriptome. Similarly, we used the program CEGMA [30], which uses a different set of highly conserved single copy orthologs to query the data sets. The preferred assembly was then run through the Evidence Gene pipeline (http://arthropods.eugenes.org/genes2/about/EvidentialGene_trassembly_pipe.html). This pipeline reduces redundancy in *de novo* assemblies by translating each contig in all six reading frames, selecting the longest open reading frame. Pairwise comparisons between all contigs eliminate all that have the same protein sequences.

### Functional annotation

We used the afterParty (https://github.com/mojones/AfterParty2) interface to annotate all of our contigs using BLAST to the Uniref90 database [31]. Canonical immune system genes from the RNAi, Toll, IMD and JNK pathways were manually annotated within, and are accessible at, the afterParty *P. interpunctella* database (http://afterparty.bio.ed.ac.uk/study/show/2194070). Contigs with a BLAST hit to the query having an e-value hit greater than 1e-10, and covering at least 80% of the full-length transcripts (inferred based on homology) were considered to be valid candidate transcripts. All contigs were loaded into Blast2Go, annotated with the non-redundant database of NCBI, and GO terms were assigned when possible. Combined graphs of the entire transcriptome were constructed for Biological Process and Molecular Function (level 2). These results were compared with the two other lepidopteran species for which equivalent data was available, *Maruca vitrata* [11] and *Manduca sexta,* with a chi-square test [12].

### Differential expression of immune system genes

The number of times each contig in the chosen transcriptome was observed in the sequence reads was calculated using HTSeq (http://www-huber.embl.de/users/anders/HTSeq/doc/overview.html). Differential expression between *P. interpunctella* that were exposed to PiGV compared to controls was calculated using DESeq (version 1.9.4) [32]. All genes (contigs) with an FDR correction of less than 0.10 were considered to be significant.

### q-RT-PCR

We analysed the expression of comp6230_c0_seq1 (cuticle protein) and comp2004_c0_seq1 (GNBP) using comparative C_T_ (ΔΔC_T_) qPCR with actin as the internal control gene. Using the RNA samples prepared for RNASeq, we synthesised complementary DNA (cDNA) by first mixing 500 ng total RNA with 500 ng random hexadeoxynucleotides (Promega) and heating to 75°C for 10 minute. The samples were chilled on ice after which 200 u MMLV reverse transcriptase (Promega), 5 μl 5x MMLV reverse transcriptase buffer, 1.25 μl dNTPs (Promega; final concentration 0.5 mM) and 20 u RNAsin RNase inhibitor (Promega) was added and the volume adjusted to 25 μl with nuclease free water. Samples were incubated at 37°C for 60 minutes, 70°C for 15 minutes and then stored at −20°C.

PCR was carried out using a StepOnePlus™ Real-Time PCR System (Applied Biosystems) and Fast SYBR Green Master Mix (Applied Biosystems) to monitor double-stranded DNA synthesis in combination with ROX as a passive reference dye. PCR reactions were carried out in duplicate using 7.5 *p*mol specific primers and approximately 5 ng cDNA in a total volume of 15 μl. The thermal profile for amplification was as follows: 95°C for 2 minutes, followed by 40 cycles of 95°C for 10 seconds, 58°C for 30 seconds and 60°C for 30 seconds. Primer pairs were designed by us and tested by standard curve analysis (primer sequences are detailed in Table 5). Expression of comp6230_c0_seq1 and comp2004_c0_seq1 (relative to actin expression) was analysed using t-tests to ask whether the expression (log transformed) was affected by infection treatment.

**Table 5:** **q-RT-PCR primers**

**Gene**	**F primer (5’- 3’)**	**R primer (5’- 3’)**
comp6230_c0_seq1 (cuticle)	CGG CTG GAA CTG ATT GCT AC	GTG TGG GAT GGA TGA TTG TG
comp2004_c0_seq1 (GNBP)	GAT GCG ACA CTA GAA TAG CTT GG	ACC CAT CTC AAC TCG CCT AC
comp24_c0_seq1 (actin)	GAT CTG GCA CCA CAC CTT CT	GGT CAT CTT CTC CCT GTT GG

## Availability of supporting data

The RNA-seq sequencing reads used to construct the assemblies are available in the SRA repository, http://www.ebi.ac.uk/ena/data/view/PRJEB5332

The assembled transcriptomes (and subsets therein) are available at (http://afterparty.bio.ed.ac.uk/study/show/2194070)

## Abbreviations

BVs: Baculoviruses, differentially expressed: differential expression
JAK-STAT: Janus kinase-Signal Transducer and Activator of Transcription
IMD: Immune deficient
PiGV: *Plodia interpuntella* granulosis virus
PxGV: *Plutella xylostella granulovirus*
UCO: Ultra-conserved orthologs
RNAi: RNA interference
GO: Gene ontology
GNBP: Gram-negative binding protein
TE: Transposable element
FDR: False discovery rate
PM: Peritrophic membrane
